# Green Routes for Bio-Fabrication in Biomedical and Pharmaceutical Applications

**DOI:** 10.3390/pharmaceutics15061744

**Published:** 2023-06-15

**Authors:** Carla Serri, Iriczalli Cruz-Maya, Irene Bonadies, Giovanna Rassu, Paolo Giunchedi, Elisabetta Gavini, Vincenzo Guarino

**Affiliations:** 1 Department of Medicine, Surgery and Pharmacy, University of Sassari, Via Muroni 23/a, 07100 Sassari, Italy; 2 Institute of Polymers, Composites and Biomaterials, National Research Council of Italy, Mostra d’Oltremare Pad. 20, V.le J.F. Kennedy 54, 80125 Naples, Italy

**Keywords:** natural polymers, green chemistry, electro fluid dynamics, nanoparticles, drug delivery

## Abstract

In the last decade, significant advances in nanotechnologies, rising from increasing knowledge and refining of technical practices in green chemistry and bioengineering, enabled the design of innovative devices suitable for different biomedical applications. In particular, novel bio-sustainable methodologies are developing to fabricate drug delivery systems able to sagely mix properties of materials (i.e., biocompatibility, biodegradability) and bioactive molecules (i.e., bioavailability, selectivity, chemical stability), as a function of the current demands for the health market. The present work aims to provide an overview of recent developments in the bio-fabrication methods for designing innovative green platforms, emphasizing the relevant impact on current and future biomedical and pharmaceutical applications.

## 1. Introduction

In recent years, the idea of bio-sustainability has been rapidly growing in many fields of research and industrial sectors, from biomedical to pharmaceutical ones. To date, the main challenge is to develop more sustainable products by the use of ‘green’—i.e., environmentally friendly—materials, also able to reproduce similar performances of traditional ones (i.e., synthetic). This is the route to manufacture innovative products by assuring high-quality stability, safety, and effectiveness standards. In this context, recent discoveries in the nanotechnology field are leading to the development of nanomaterials with unique physical and chemical properties in terms of tailored sizes at the nanoscale and specific surface functionalisation, such as those facilitating the effective use of green processes, either in traditional medicine or for the development of new approaches in nanomedicine [1]. 

In the pharmaceutical area, the use of raw materials from natural sources has just been demonstrated to be a valid solution to define novel routes of drug administration, maximising therapeutic benefits, in the view of well-known limitations of conventional pharmacological treatments, in terms of solubility, bioavailability, half-life, and crossing physiological barriers. Using current bio-fabrication methods in a more green vision could help design innovative systems (nanovectors, nanofibers, micro- and macro-gels, nanoparticles) which show greater respect for the environment and health demands [2,3,4]. 

In this view, strong efforts have been made to optimise physical, chemical, biological, and hybrid methods to design innovative platforms able to control drug release in target sites—by different technological methods (i.e., solvent evaporation, nanoprecipitation, ionotropic gelation, single or multiple emulsions) [1,5]. However, their use in the biomedical field seems to be often restricted by the toxic response of released molecules and chemicals used during the bio-fabrication process [6]. Hence, it is compelling the development of more reliable and eco-friendly technologies that implement the synthesis/bio-fabrication of drug-loaded carriers on two basic principles: (a) the use of benign chemicals to manipulate constituent matrices, (b) the minimisation or the removal of hazardous and chemically aggressive substances during all the manufacturing stages [7]. 

This review highlights main challenges associated with fabricating substrates/devices for pharmaceutical applications from a green perspective. Accordingly, green materials and bio-fabrication processes will be discussed in the first part of the work. The second part will present a set of innovative polymer-based formulations, emphasising their use for different green or bioinspired applications (i.e., pharmaceutical, biomedical, and nutraceutical). 

## 2. Green and Bio-Sustainable Materials

Biocompatible/biodegradable polymers have been rapidly diffused in the last two decades to fabricate a wide range of high-performance medical devices for drug delivery and medicine applications. This mainly depends upon their efficient interfacial interactions with biological matter (i.e., cells, proteins, water, ions) and the ability to control loaded drugs’ kinetic release by their degradation. They are frequently polymers derived from synthetic sources, generally characterised by hydrolysable bonds along the backbone, such as polyesters, polyurethanes (PUs), polyanhydrides, and vinyl polymers. They also include FDA-approved materials such as poly(lactic acid) (PLA), poly(glycolic acid) (PGA) and their copolymers (PLGA) and Poly(ε-caprolactone) (PCL), widely investigated for the design and fabrication of drug delivery systems, due to their attitude to degrade into biocompatible monomers by hydrolytic reaction in physiological conditions [8,9]. 

However, most parts are not bio-sustainable, being derived from raw materials (i.e., petroleum based). An alternative concerns using naturally derived materials considered green for definition. Overall, they include biopolymers from animals (i.e., hyaluronic acid, chitosan, collagen, gelatin, elastin, albumin, silk [10,11]) or vegetable (i.e., arabic gum, algae such as alginates, microbials such as dextrans [10,12]). However, their process is more complex, thus requiring, to a large extent, further pre-processing steps (i.e., extraction, purification) to obtain the final product, with relevant effects in terms of high production costs and limited yield. 

In this view, the real challenge is identifying biomaterials obtained from renewable re-sources that are economically sustainable for environmental and human health. Nowadays, more sustainable development levels have to be reached, and one of the ways is to contribute to reducing the environmental impact generated by the current polymer industry. The ongoing research on sustainable polymers in terms of new materials paves the way to introducing green materials—i.e., derived from nature or biomass. 

Bio-sustainable polymers such as starch, cellulose, hemicellulose, lignin, keratin, and silk fibroin have been used since ancient times thanks to their huge availability and properties; currently, they are still studied and used because they exhibit cost-effective properties and are biodegradable and biocompatible. In some cases, they can also be obtained by biomasses from industrial wastes. For instance, plant biomass is renewable and can be created in relevant quantities, comprising various components, such as cellulose, hemicelluloses and lignin, wood, and agricultural residues. Alternatively, other industrial processes, such as food processing, can furnish biomasses suitable for bio-based applications [10].

Cellulose is the most abundant biopolymer in the world; it can be found in plants and biomasses and used in different application fields thanks to its chemical–physical properties. Cellulose macrofibres are composed of microfibrils formed from nanofibrils with a crystalline part and an amorphous part in a row. Cellulose nanocrystals (CNCs) and nanofibers (CNFs) have been used as alternative green and bioactive fillers in a broad field of innovative nanostructured materials [13,14,15,16]. Lignin is the second most abundant biopolymer intimately intertwined with hemicellulose, forming a matrix surrounding the ordinated cellulose microfibrils. These components can be easily obtained as fibrous by-products of wild processing; for example, pecan nuts are reported in the literature as a source of fibres for polymer and composite materials for packaging applications [17]. Additionally, durian—whose eaten part is only one-third of the whole fruit—is an ideal cellulose resource for natural hydrogel dressing preparation [18].

Chitosan is derived from the deacetylation of chitin, the second most plentiful polysaccharide in nature after cellulose, typically found in the exoskeletons of arthropods and crustaceans and also in fungal cell walls. It is a polycationic polysaccharide containing free amine groups in neutral or basic media, whereas protonated amines are in acidic media. Thanks to these pH-sensitive features and its rigid chemical structure, chitosan can form films, gels, and microcapsules useful for controlled drug release [19,20].

Among protein-derived polymers, zein comprises almost 80% of the whole protein content in corn, one of the most abundant agricultural cultivars. It has attracted significant interest from the scientific community thanks to its broad presence worldwide and chemical/physicochemical properties such as non-toxicity, biodegradability, processing versatility, film-forming ability, low water vapour permeability, grease/oil proof, and biocompatibility. It is well known that proteins may carry different charges depending on the pH due to the presence of amino and carboxyl groups; for this reason, zein can be considered a promising candidate to efficiently encapsulate active compounds such as propolis or micronutrients [21]. 

Silk fibroin is the fibrous protein produced by several insects and arachnids. Still, the ease, availability, and cost-effective nature of harvesting silkworm cocoons have made Bombyx mori silkworm cocoon the main source for centuries. It comprises various amino acids, including alanine and glycine. They can be mixed with other proteins, such as Sericin [22], to improve biocompatibility, tunable biodegradability, and mechanical strength. Thanks to the use of novel bio-fabrication processes, an inflammatory response associated with their combined use is partially solved, giving the opportunity to successfully use them for regenerative applications, additive manufacturing, sustainable devices, controlled release systems, dermal fillers, skincare, haircare, cosmetics, bio-surfactants, and textiles [10,23].

Byssal threads of mussels resemble the silk of spiders or silkworms in many aspects. Like silkworm silk, which consists of anisotropically dispersed fibres (fibroin) in a sticky resin (sericin), byssal threads are composed of collagen fibres dispersed in the polyphenolic resin (3,4-dihydroxy-Lphenylalanine—L-DOPA). L-Dopa is also a crucial intermediate for the biosynthesis of several compounds, including polydopamine. Its oxidative polymerisation produces melanin and can be mimicked to prepare biomaterials such as eumelanin and pheomelanin. So far, several papers have been published on mussel-inspired polymers used as hydrogels for nanomedicine and tissue engineering, environmental applications, adhesives, and antifouling coatings [4,24,25,26].

Bio-sustainable materials can also be partially composed of green and synthesised polymers or obtained from biological resources but polymerised with traditional processes, for example, polylactides derived from corn sugar, polyols from sugar-alcohols, polyamides from castor oil, and polyhydroxyalkanoates (PHAs/PHBs) from microorganisms [27].

PLA, the most abundant aliphatic thermoplastic polymer, is made from renewable sources such as sugarcane, maise, cassava, corn, etc. Its advantages include being inexpensive, environmentally friendly, easy to produce, recyclable, compostable, and biocompatible; furthermore, degradation products of PLA are also non-toxic to humans and the environment. By varying the molecular weight and degree of crystallinity and the stereo-chemical configuration of the molecular structure of the PLA backbone, it is possible to tune physical and mechanical properties. It is elementary to process PLA using different techniques and combine it with various fillers and additives to overcome its drawbacks and add specific functionalities. For this reason, it has many applications in other fields ranging from packaging to nanomedicine. For example, it has been used to realise controlled drug delivery systems or scaffolds for tissue engineering [28,29,30,31,32,33,34,35,36].

Polyhydroxyalkanoates (PHAs) are a class of biodegradable linear polyesters consisting of hydroxyalkanoic acid monomers produced in nature by the action of bacteria during the fermentation of sugar or lipids in famine conditions. PHAs represent an excellent alternative to conventional polymers in the frame of the circular economy since they are fully bio-based and biodegradable also in the marine environment; the physical and chemical qualities such as biodegradability, and biocompatibility that stimulate the numerous cellular processes governing cell adhesion, proliferation, migration, and plasma stability, as well as the cellular activity in tissue [37]. However, the lengthy synthetic processes and expensive manufacturing methods restricted its supply. 

Among the different commercially available PHAs, the isotactic homo-polyester poly(3-hydroxybutyrate) (PHB) and, above all, its copolymer with 3-hydroxyvalerate (HV)-poly(3-hydroxybutyrate-co-3-hydroxyvalerate) (PHBV) can prove advantageous for different applications since it shows higher ductility, reduced crystallinity, and lower Tm. Considering specific modifications, PHAs also hold great potential for developing scaffolds for tissue engineering and medical devices (stents, grafts, pins, patches, etc.) suitable for drug delivery and implants (Table 1) [38,39,40,41,42].

## 3. Bioprocessing Techniques

Using green biomaterials as a viable alternative to fossil-based biomaterials opens many opportunities to process them via eco-friendly processing technologies. In recent years, a significant emphasis was mainly addressed on chemical methods based on green-inspired reactions (i.e., sol–gel technology, redox processes, pyrolysis, microwave, photo-chemical/electrochemical, hydrothermal) able to satisfy some sustainability issues posed by conventional synthesis methods [52,53]. This facilitates the development of different typologies of nanoparticles—i.e., basically made of inorganic materials—exhibiting bio-accumulative or toxic features that limit their applicability in the biomedical field, mainly due to reduced reproducibility of the final products and limited stability in biological fluids [54,55].

Recently, it is forcefully emerging a wide range of processing technologies involving greener solvents that are characterised by lower vapour pressure, higher thermal stability, better bonding abilities and lower toxicity compared with conventionally used solvents [56]. Among them, ionic liquids (ILs) have been variously adopted in biopolymer processing for pharmaceutical applications due to their ability to dissolve hydrogen bonding among chains, not affecting the molecular weight. Indeed, ILs are suitable not only for biopolymer dissolution but also for the co-dissolution or co-dispersion of pharmaceutical actives in composite matrices with different functionalities [57,58].

This approach is currently used to process a wide range of bio-based polymers, including polysaccharides (i.e., chitin, chitosan), proteins (i.e., silk keratin, starch, lignin), and more [15,59] for the fabrication of different substrate typologies, i.e., porous matrices, scaffolds, composite membranes, microbeads, and filters.

**Figure 1:**
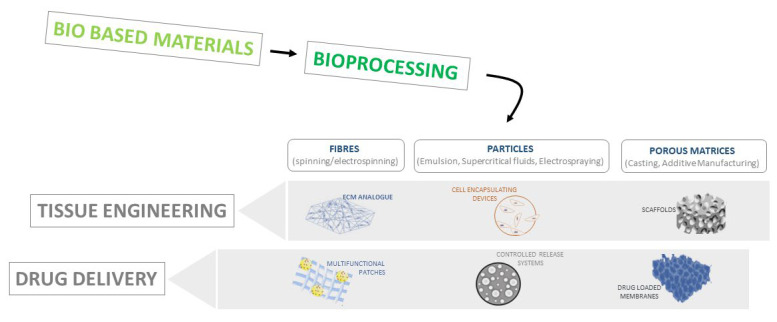
The basic scheme of bioprocessing techniques suitable for fabricating devices for tissue engineering and drug delivery.

In this view, different processing techniques traditionally used to process synthetic materials are currently receiving a strong interest for their ability to be adapted to many green materials. They include emulsions, dip coating, 3D printing, supercritical fluid technologies, electrospinning, and electro fluid dynamic processes [60,61,62] (Figure 1).

The most common method for preparing drug-loaded systems is the emulsion that facilitates precipitating polymers as micro/nanoparticles by solvent evaporation or solvent–non-solvent exchange. Traditionally, it involves the mixing of two or three different phases, constituted by polymers/actives dissolved/dispersed in immiscible solutions (i.e., single (O/W) or double emulsion (O/W/O)). Accurate control of the mixing conditions (i.e., mechanical or magnetic stirring, sonication) allows us to control the size of dispersed phases. At the same time, the use of surfactants stabilises the interface between dispersing and dispersed phases [63] (Figure 1). 

Several studies have investigated using ILs to fabricate green micro-emulsions from bio-based polymers (PLA, cellulose) [64]. The first attempts have been addressed to employ ILs to replace the water in the dispersing phase [65]. More recent studies have demonstrated the efficient role of ILs as organic solvents able to solubilise in oil-based surfactants, thus facing the main requirements of pharmaceutical applications [66]. Indeed, ILs can be successfully used to solubilise a range of compounds and actives poorly soluble in water: beyond their innate capability to be easily dissolved in water due to their ionic character, some ILs possess hydrophobic groups (i.e., alkylated, fluorinated) that make them hydrophobic and immiscible with water, thus promoting weak interactions with the solute [67,68]. Accordingly, different technological strategies were optimised to entrap bioactive molecules by coagulation/precipitation mechanisms mediated by polar interactions. For instance, from 5 microns to 1 mm, porous microbeads were fabricated by chitosan-derived biomaterials by water precipitation [69]. Because of their poor solubility under physiological conditions, chitosan beads tend to swell in water. The use of ILs improve the solubility of the polymer, also supporting the entrapment of drugs by chitosan chain interactions. An alternative approach consists of stabilising nanoparticles surface by creating functional coatings (i.e., cellulose) that can act as a barrier to molecular diffusion and provide available sites for their functionalisation [70]. In this case, beads were coated with cellulose dissolved in the ionic liquid (i.e., 1-ethyl-3-methylimidazolium acetate). As water adsorbed by the chitosan network meets cellulose solution, water is rapidly stripped by the polar interactions with ILs. Accordingly, entrapped drugs loaded in the chitosan core can be released by tuning kinetics as a function of the environmental conditions (i.e., pH), able to influence local polar interactions. 

More recent studies explore combining non-volatile polar fluids such as ILs with volatile non-polar ones—i.e., scCO_2_—as an efficient pharmaceutical and food industry strategy. Indeed, it is well known that scCO_2_ is highly soluble in ILs, while ILs cannot easily dissolve in scCO_2_. This allows the diffusion of drugs/actives (generally soluble in scCO_2_) from the ILs-phase into the scCO_2_ phase [71]. Hence, various approaches based on supercritical fluids have been investigated to find a technological solution for improving drug encapsulation into powder-like particles (i.e., mean size < 500 nm) suitable for inhalation by the nose to the lungs or for other delivery routes (i.e., oral, intravenous, ophthalmic, transdermal, and implants) [72].

It is noteworthy that the use of supercritical fluids methods (SCFs) such as Rapid Expansion of Supercritical Solutions (RESS) or Supercritical Anti Solvent (SAS)—not requiring the use of Ils—even allows us to dissolve bio-based polymers in the place of organic solvents, providing efficient strategies for the fabrication of more sustainable pharmaceutical devices, i.e., those that also address the minor consumption of toxic chemicals for use in health and environmental care scenarios [63]. RESS can be used for soluble CO_2_ molecules, while SAS can deal with non-soluble molecules [32,73,74]. The main advantage of both methods is that they are suitable for continuous operation, mainly designed for industrial production. SCF technology is one of the most effective substitutes for environmental friendliness, the convenience of processing, and the economically promising nature of SCFs. The outstanding characteristics of SCFs have enabled the fabrication of various active pharmaceutical ingredients (APIs), alone or in combination with compatible supramolecular species, to achieve improved drug processes, enabling us to obtain systems with a pharmaceutical activity that meets the prerequisites of green nature [7].

Electro-fluid-dynamic techniques (EFDs) are emerging as highly flexible and low-cost processes to manipulate polymers and drugs in solution by applying electrostatic forces generated via high-voltage electric fields [75,76]. They include a pattern of “bottom-up” technologies such as electrospinning or spraying and their combinations, showing a high process versatility, not limiting the opportunity to control the characteristic size del carrier accurately—from micro- to sub-micrometric size scale—making them suitable for manufacturing of highly scalable devices in the form of particles or fibres or their assemblies, for research use or industrial processes [77]. Electrospinning—the most diffused among the EFDs—is considered the most consistent technology for fabricating polymer or composite fibres with accurate control of size scale—from several micrometres to nanometres. In this case, applying electric forces generated by a high-voltage electric field is pivotal to overcoming the surface tension of the viscous solution needed to form the polymer jet and, ultimately, the fibres. Indeed, polymer droplet is interested in the antagonism of three different forces: columbic, viscoelastic, and surficial. By applying an increasing voltage, electric forces increase, until reaching the threshold value that allows it to overcome the other ones, thus promoting the ejection of a polymer jet and the formation of fibres [75,78]. In this context, a large range of factors over the voltage plays a relevant role in this mechanism, including operative parameters (i.e., flow rate, collector distance), polymer, solvent/co-solvent chemistry, or environmental conditions (i.e., humidity, temperature) [79,80].

However, electrospinning is drastically limited in sustainability by using environmentally unfriendly solvents characterised by low boiling points, i.e., chloroform, that assure the mechanism of fibre formation by evaporation. For this purpose, an interesting strategy is represented by the use of melt electrospinning to avoid the use of solvents [81]. However, this requires using polymers over the melting temperature, which can be reasonably incompatible with the stability of most active molecules used for drug delivery applications. Only a few studies have demonstrated the suitability of melt electrospinning to fabricate Eudragit^®^-based fibres to encapsulate poorly water-soluble molecules [82]. As for the fabrication of medical devices for drug release, electrospun fibres need to be processed by the use of benign solvents able to preserve the stability of a wide variety of molecules and actives to preserve their therapeutic functions.

Meanwhile, biopolymers must provide all the chemical/physical cues deputed to control active transport [83] or adsorption mechanisms [84] that mainly address molecular release by diffusion mechanisms. For these purposes, a wide range of processing methodologies were proposed to fabricate drug-loaded electrospun fibres mainly based on post-spinning chemical modifications of the fibre surfaces or the optimisation of the experimental setup (i.e., emulsion spinning [85], core–shell electrospun fibres [86]) suitable for improving the control of molecular release mechanisms in time and space. More recently, additive methodologies integrating chitosan nanoparticles—processed via electrospraying [87,88,89]—into a fibrous network have demonstrated a highly versatile route to more efficiently functionalised polymer matrices by bioactive signals and molecules [90]. This integrated technological approach can allow us to design multicomponent devices, with different structural properties and molecular release, as a function of the complexity degree of the production process.

**Figure 2:**
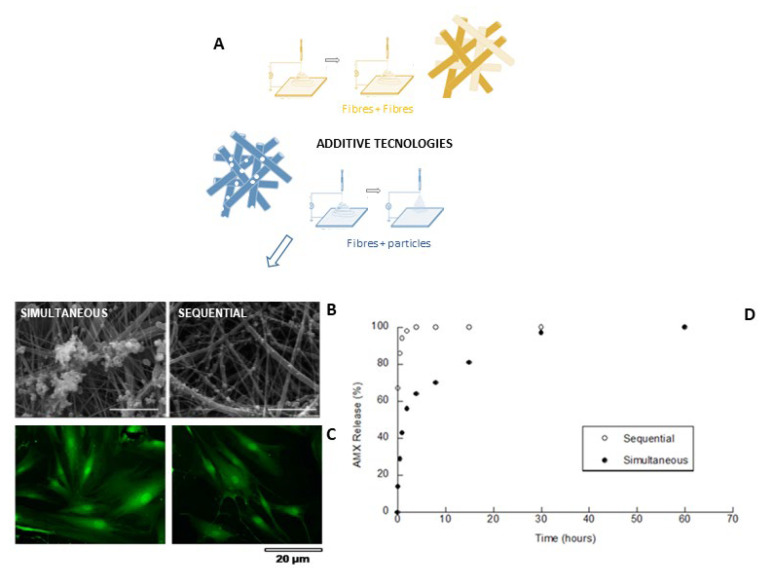
Additive electrospinning/spraying: schematic description of the process to collect chitosan nanoparticles onto fibre assemblies (**A**) and comparison between simultaneous and sequential deposition mode: (**B**) morphology [87], (**C**) in vitro hMSC response, and (**D**) molecular release profiles [91] (copyright requested).

The large customisation of the process also can allow switching among different modes to integrate nanoparticles into the fibrous network (i.e., sequential [91], simultaneous [92]), giving a chance to more accurately match release mechanisms to the specific applicative demands (Figure 2). 

A sister technological approach is based on the use of electrical forces to manipulate biopolymers such as chitosan in the form of microcapsules, allowing us to overcome some criticisms of conventional technologies in terms of encapsulation efficiency, limited process scale up, poor control of particle size (lower that 100 micron), non-homogeneous size distribution [93].

**Figure 3:**
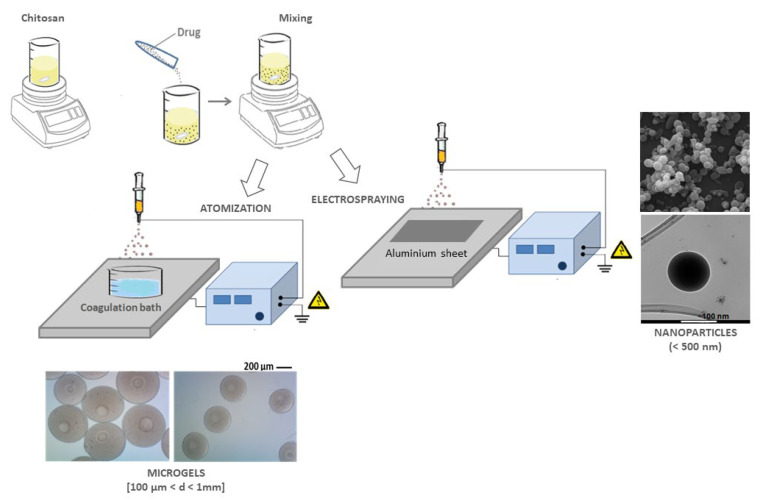
Drug-loaded chitosan capsules via Electro Fluid Dynamic Atomization (EFDA) and electrospraying (ES): scheme of preparation and particle morphology (adapted from [94]).

As schematised in Figure 3, the working principle is based on the ability of electric forces to charge, deform, and disrupt an aqueous polymer solution into small droplets by the competition between Coulomb forces related to surface charge and cohesive forces inside the droplet without the use of additive forces (i.e., mechanical) to induce the polymer atomisation. Some differences can be recognised in the experimental setup typically used in EFDA and ES processes (Figure 3), as confirmed in previous studies [94,95]. Pure ES is a continuous process working at low flow rates—from 0.1 to 0.3 mL/h—to rapidly reach the excess of charge density once the solvent evaporation rapidly occurs. Conversely, EFDA is a non-continuous process based on the dripping of spherical droplets—by applying high flow rates—from 1 to 5 mL/h. In this case, the solvent evaporation rate is insufficient for particle precipitation. Still, chemical/physical strategies are needed to remove any solvent traces and ensure the formation of spherical particles. It is noteworthy that no excess in charge occurs in this case, but only particles—several hundred microns in size—can be obtained. Recent studies have demonstrated a large versatility of both techniques, both suitable for a sustainable molecular delivery, due to the opportunity to easily adapt the working principles to a wide range of biopolymers and solvents—even water to some extent—without significant limitations in terms of processability [96].

## 4. Pharmacological Species

### 4.1. Active Pharmaceutical Ingredients (APIs)

The classification of drugs is extensive and complex because of increases in new molecules. The development of new scientific technology discovery has significantly increased pharmacologically active molecules due to computer-assisted drug design, further screening, and combinatorial chemistry. This is all due to the discovery of new drugs required for the increase in new specific pathological diseases [97]. In recent years, the new study of pharmacokinetics processes has led to further clarifications of the mechanisms of action among drugs. It turns out that the combination of chemotherapeutics and natural substances is very interesting; it was found that the combination of gemcitabine and quercitin causes a synergistic effect by inhibiting metastatic pancreatic cells, decreeing quercitin, a possible candidate as a metastatic agent [98]. In fact, the goal of chemotherapy drugs is to inhibit the proliferation of cancer cells while trying to prevent metastasis formation. Standard therapy by chemotherapy leads to toxic effects because the drug acts on normal cells [99]. Therefore, multiple drugs can be combined for clinical treatments [100]. Active pharmaceutical ingredients (APIs) currently represent a fundamental resource for human health.

API compounds exhibit different physical and chemical properties and bioactivity; solid APIs have many drawbacks, including limited solubility, poor bioavailability, and polymorphic conversion. The classification of these substances is of great interest to the pharmaceutical industries. The solubility and poor solubility of the molecules determine the variation in classification. In particular, in vitro dissolution studies or in vivo permeations studies have been carried out [101]. Specifically, using these drugs that are produced using safe methods is always of great environmental interest. These methods must have a substantial impact on both human health and the environment. However, after the patient’s use, their effect on the environment (exposed wildlife, water contamination) is becoming a relevant problem that requires substantial efforts to define new rules from governments. This leads to great interest in improving the activity of these molecules by encapsulating them in nanoparticles. It turns out to be interesting that APIs worldwide classification encompasses several classes of drugs. In brief, these classes of drugs are antibiotics, chemotherapeutics, antidiabetics, antioxidants, anti-inflammatory, cardioprotective, and nutraceuticals [7].

### 4.2. Actives from Natural Products 

There are a wide range of agents derived from nature, many of them still unexplored, able to fulfil all the current needs. Among them, some active agents are gaining relevant interest for pharmaceutical applications. In addition, natural products often ensure better tolerability and bioavailability, and their pharmacological activities can affect the immune system in multiple ways.

Plants are the most abundant source of active agents; medicines derived from plants have played a pivotal role in the health care of many cultures, above all in India and China. Traditional Chinese medicine, used for thousands of years, mainly uses plants and minerals. Still, traditional Chinese medicine often inspires drug design and development [102,103].

Turmeric—a product of *Curcuma longa*, a rhizomatous herbaceous perennial plant—has a long history of medicinal use, with more than 100 components having been isolated from the spice. The main components of the root are a volatile oil containing turmerone and the yellow pigments curcuminoids—chemically related to its principal ingredient, curcumin, a polyphenol—both of which have been revealed by traditional and modern medicine as having potent antioxidant, anti-inflammatory, antimutagenic, antimicrobial, and anticancer effects. As reported in the literature, curcumin has diverse molecular targets such as transcription factors, growth factors and their receptors, cytokines, enzymes, and genes regulating cell proliferation and apoptosis, influencing numerous biochemical and molecular cascades [104,105]. 

Phenolic compounds embrace polyphenols (tannins and flavonoids), simple phenols (phenolic acids) and other phenols such as lignans, and coumarin stilbenes, ranging from simple to conjugated complexes; they bear one/more aromatic rings with one/more hydroxyl moieties and, thanks to hydroxyl groups located on the benzene rings, have strong antioxidant activity. The ability of species to act as antioxidants concern the delay or control of oxidative processes triggered by free radicals, reactive oxygen species (ROS), reactive nitrogen species (RNS), and other oxidants. They are also potent inhibitors for several enzymes, such as xanthine oxidase (XO), cyclo-oxygenase (COX), lipoxygenase, and phosphoinositide 3-kinase. Flavonoid subgroups generally have nutritional properties and therapeutic potential on different pathologies, such as cancer, cardiovascular, neurological, inflammatory, and metabolic diseases. Is widely reported the application of flavonoids in skin diseases for the prevention and treatment of photo-ageing, skin cancer, and wound healing but also in other types of cancers, thanks to their ability to decrease inflammation marker proteins, as well as pro-apoptotic proteins and autophagy markers as revealed in in vitro studies [106,107,108,109].

In addition to curcumin, there are many other flavonoids extracted from plants. Currently, there are about 6000 flavonoids that contribute to the colourful pigments of fruits, herbs, vegetables, and medicinal plants. For example, anthocyanins—one of the six subgroups of flavonoids—are the pigments in the outer cell layers of various plants, flowers and fruits responsible for their colour, such as in cranberries, black currants, red grapes, merlot grapes, raspberries, strawberries, blueberries, bilberries, and blackberries [107]. Quercetin (3,3′,4′,5,7-pentahydroxyflavone) is widely distributed among a diversity of vegetable species, e.g., cabbage, broccoli, tomatoes, peppers, asparagus, etc., and medicinal plants, e.g., *Ginkgo biloba*, *Hypericum perforatum*, and *Sambucus canadensis*. Thanks to their properties, flavonoids, especially quercetin, are used in combination with delivery nano-vectors to have a controlled drug delivery for wound healing [33] and the regeneration and functional recovery of the nervous system. [109]. Resveratrol (3,4′,5 trihydroxystilbene)—found in products commonly consumed in the human diet, such as red wine, grapes, and peanuts—can prevent or slow the progression of a wide variety of illnesses, including cancer, cardiovascular disease, and ischemic injuries, as reported by several papers [110]. Many resveratrol nano-formulations are reported in the literature because of their poor aqueous solubility and low bioavailability [20,111]. Among the phenolic acid compounds, we can notice gallic acid (3,4,5-trihydroxy benzoic acid), found abundantly in tea, grapes, berries, and other fruits as well as in wine [112]; caffeic acid (3,4-dihydroxycinnamic acid), present in many food sources, including coffee drinks, blueberries, apples, cider, and propolis [113]; rosmarinic acid, a dimer of caffeic acid ((R)-α-[3-(3,4-dihydroxyphenyl)-1-oxo-2 E-propenyl]oxy]-3,4-dihydroxy-enzenepropanoic acid), isolated for the first time in 1958 from the rosemary plant [114]; and tannic acid, a natural hydrolysable polyphenol composed of ten gallic acid molecules commonly purified from the gallnuts produced by some species of oak and sumac trees [115].

A very plentiful source of natural extracts is a honeybee colony; it produces various products, such as honey, bee pollen (BP), bee bread (BB), royal jelly (RJ), propolis, beeswax, and bee venom. Honey is well known for its nutritional contents. Still, all bee products are rich in natural extracts such as polyphenols, proteins, vitamins, and enzymes that can vary in proportion according to the geographical origin of the colony. Thanks to this wide range of ingredients, honeybee products are massively used for different application fields: propolis has demonstrated antimicrobial activity in clinical, in vivo, and in vitro studies; bee venom can protect dopaminergic neurons from degeneration in experimental models of Parkinson’s disease; RJ has antioxidant activity and hepatoprotective effect; bee pollen has anti-atherosclerotic and cardioprotective activity and a significant effect on metabolic syndrome disorders. For this reason, all these products are encapsulated or mixed in nano-vectors to have a controlled and topic delivery of active ingredients [116,117,118,119].

Complementary to phenolic antioxidants, also vitamins are widely used as active ingredients. Vitamin E—α-tocopherol—is considered the most potent natural lipophilic antioxidant, enabling it to fight lipid peroxidation in cells through chain-breaking reactions [106]. Recently, much attention has been focused on vitamin E succinate (VES)-based nano-carriers since, combined with other drugs, they can inhibit tumour cellular proliferation. They can be utilised as an antineoplastic agent in the clinic [120]. Delivery systems that could also carrier vitamin C and increase its solubility, stability, bioavailability, and predisposition to traverse epithelial barriers have been developed [121]. 

## 5. Applications 

### 5.1. Pharmaceutical Applications: Drug Release and Cancer Therapy

Generally, standard chemotherapy produces toxic effects because the drug acts on normal cells. The main trouble for drugs is crossing barriers due to differences between the cell connections or chemical–physical processes. Therefore, all mechanisms available to overcome barriers must be evaluated. The pharmacokinetic problems described in the previous section have led to the emergence of nanoparticulate systems to improve the solubility and efficacy, bioavailability, and half-life of many drugs [99]. Controlled or targeted drug delivery systems have been developed to resolve a wide range of pharmacokinetic severity. Through the use of these carrier systems, it is easier for different barriers to be crossed that exist throughout the body tissues. Thus, there are many essential elements to consider when designing nanoparticles for drug delivery to overcome specific barriers [122]. The purpose of developing these nanoparticle systems is for the drug to cross a barrier without changing the pharmacokinetics of a conventional dose [123]. Engineered nanoparticles have proven attractive in pharmaceuticals for drug delivery, diagnostics, and nutraceuticals. PLGA is one of the synthetic polymers used in the pharmaceutical field. Interestingly, using these biocompatible polymeric materials with human tissues can increase the efficacy of poorly soluble drugs. These nanoparticles may have a functionalised surface that allows them to encapsulate or transport drugs and proteins at the target site. The drug can be used at a 10–500 nm nanoscale carrier. The materials characteristics used to prepare engineered nanoparticles vary; this variation is given by the different materials used, whether lipid or polymeric. In contrast, in polymeric materials, interactions are investigated due to the chemical properties of the drug and the materials used. Therefore, biodegradable materials must be used when using polymeric materials for transporting and targeting drugs. A nanoparticle system must have a therapeutic, diagnostic, and curative effect. The main objectives of using a nanoparticle system for the administration of drugs should be:

- Targeted release to the site;
- More drug release with more significant therapeutic effect;
- Reduced toxic effects;
- Increase safety and biocompatibility;
- Preparation and development of new medicines.

**Figure 4:**
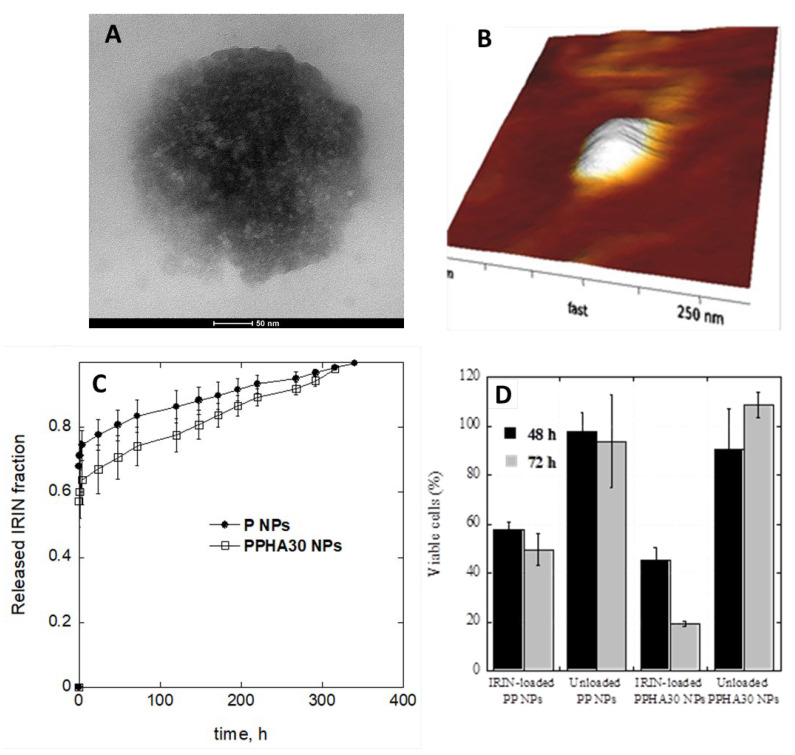
PLGA nanoparticles (NPs) were decorated with hyaluronic acid (HA) moieties. Structure of polymeric nanoparticle-based targeted drug delivery system. Selected TEM (**A**) and AFM (**B**) micrographs PPHA (NPs); in vitro IRIN release profiles from NPs (**C**) and results of cytotoxicity assay was calculated with respect to the non-treated control cells (**D**) [128] (copyright requested).

Targeted drug delivery using nanomaterials versus free drugs is utilised in clinical cancer therapy. NPs can be used for different routes of administration, as described in the previous chapter. NPs used for the parenteral route of administration are aimed at the indirection of the drug to specific tissues. The goal of targeted therapy is based on targeting specific cancer cells using passive targeting or active targeting. The enhanced permeability and retention (EPR) effect is found in passive targeting, while active targeting is influenced by material-conjugating with antibodies, peptides, aptamers, and small molecules. Compared with free drug administration, the drug can improve its solubility, half-life times, biocompatibility, and loading capacity and reduce toxic effects [124,125,126]. Cancer-induced angiogenesis produces many immature vasculatures that repress lymphatic drainage; diameter small NPs can exploit the EPR effect to direct the drug to the level of tumour lymphatic vessels [127]. Binding peptides or using a hyaluronic acid coating to direct a particle to the target site level is interesting. Hyaluronic acid (HA) is very interesting in the pharmaceutical field. It binds to the CD44 over-expressed receptor at the level of tumour tissues. In this work, we report a spontaneous arrangement of HA on the surface of Irinotecan (IRIN)-loaded poly (lactic-co-glycolic acid) (PLGA) nanoparticles through a modified single emulsion–solvent evaporation method (Figure 4).

**Figure 5:**
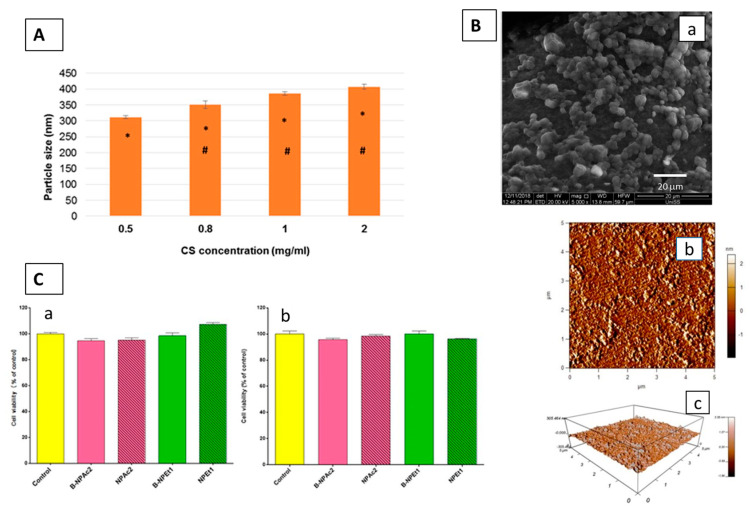
Intranasal Delivery of Genistein-Loaded Green nanoparticles. (**A**) Influence of chitosan (CS) concentration on the particle size, * *p* < 0.05: 0.5 vs. 0.8, 1, and 2; # *p* < 0.05: 0.8 vs. 1 and 2; (**B**) SEM pictures of pure drug (a) (scale bar: 20 μm), topographic images of purified chitosan (b) and of loaded nanoparticles (c) (scale bar of 5 × 5 μm scan); (**C**) effects of two different chitosan unloaded (B-NPAc2 and B-NPEt1) and loaded (NPEt1 and NPAc2) nanoparticles prepared in acetone (pink bars) and ethanol (green bars) on PC12 cells viability after 24 h of exposure, evaluated by MTT assay (a) and trypan blue assay (b) [131].

Nab-paclitaxel is an example of a clinical drug for treating several cancers. The technology used for nab-paclitaxel uses albumin to deliver paclitaxel with an attractive pharmacokinetic profile. Despite the clinical benefit of solvent-based (sb) taxanes, these agents can be associated with significant and severe toxicities. Albumin-bound paclitaxel (Abraxane; nab^®^-Paclitaxel), a novel solvent-free taxane, has demonstrated higher response rates and improved tolerability compared to solvent-based formulations in patients [129,130]. Among the routes of administration to direct drug delivery, nasal delivery has gained much scientific attention. In these systems, improving the delivery of some drugs can cross the blood–brain barrier (BBB) with certain carriers [125,131]. This study aimed to design and characterise genistein-loaded chitosan nanoparticles for intranasal drug delivery, prepared by the ionic gelation technique using sodium hexametaphosphate. The endothelial barrier is very narrow and impenetrable; these problems can be solved using carriers that mask some of the issues the free drug would run into, such as P-glycoprotein interaction. P-glycoprotein is one of the ATP-dependent transporters that has a critical essential physiological role in blocking the entry of certain drugs into the brain and may be highly expressed in drug-resistant cancer cells. A study was designed to characterise genistein-loaded chitosan nanoparticles for intranasal drug delivery, prepared by the ionic gelation technique using sodium hexametaphosphate (Figure 5). Several studies suggest that loading Paclitaxel into nanoparticles improves drug uptake in the cerebellum since the step that would see the drug being blocked by the glycoprotein-p is bypassed. Thus, it is very attractive for the entrapment of chemotherapeutics such as paclitaxel, doxorubicin, cisplatin, irinotecan, and gemcitabine.

**Figure 6:**
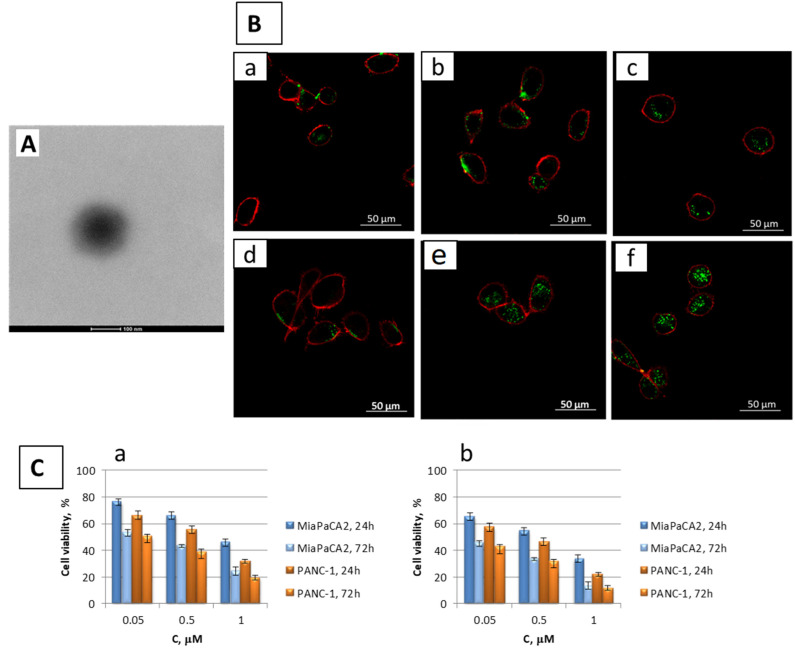
Hyaluronic acid-decorated nanoparticles loaded with quercetin and gemcitabine for the combination therapy for the treatment of pancreatic cancer. (**A**) Representative TEM images of HA-coating NPs PPHA (**B**) Uptake images, obtained by confocal microscopy, of MiaPaCa-2 cells (5 × 10^3^ cells/well) after 0.5 (a,d), 8 (b,e) and 24 h (c,f) of incubation with fluorescent PP (a–c) or PPHA NPs (d–f) at 0.3 mg/mL. Green signal: fluorescent PPNPs and PPHA NPs; Red signal: cellular membrane labeled with tetramethylrhodamine-conjugate concanavalin A. Scale bar: 50 µm; (**C**) Viability of Mia Pa-Ca2 and PANC-1 cells, after exposure to the combination of GMC and CD/QCT: free GMC NPs + PP_CD/QCT NPs (a); free GMC NPs + PPHA_CD/QCT NPs (b); (from [98]).

The combination of gemcitabine (GMC) with quercetin (QCT) shows a synergistic effect in inhibiting the migration of pancreatic cancer cells. Consequently, herein, GMC and QCT have been loaded within biodegradable NPs based on poly(lactic-co-glycolic acid), externally decorated with hyaluronic acid (HA; viz., PPHA NPs), which plays a major role in drug targeting to cancer cells due to its ability to specifically interact with CD44 receptor, which is overexpressed in many cancers (Figure 6) [98,129]. This determines that drug passage and absorption can be improved in all routes of administration used and toxic effects reduced by targeting the desired target site.

One of the more productive green methods is certainly based on employing supercritical fluids, not requiring organic solvents for polymer dissolution, with remarkable benefits on both environmental and pharmaceutical aspects. For this reason, several research groups are testing SCF technology for fabricating new pharmaceutical carriers loaded with inorganic or nutraceutical substances (i.e., CoQ10, vitamins) of growing interest in the pharmaceutical field [7,71].

**Figure 7:**
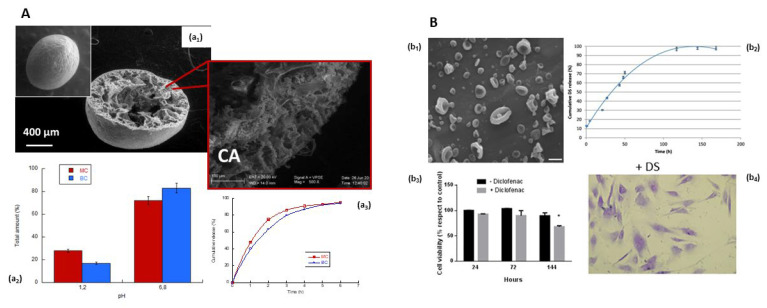
Green particles for oral administration. (**A**) Core–shell microparticles of chitosan/cellulose: (a_1_) morphology; (a_2_) encapsulation, and (a_3_) in vitro release of ketoprofen—SGF (pH 1.2) for first 2 h and SIF (pH 6.8) for 6 h [133]. (**B**) Cellulose-graft-poly(ε-caprolactone) nanoparticles obtained by electrospraying with differences in the morphology where the inclusion of drugs influences the polar interactions with grafted chains of polymer in solution (b_1_), while the in vitro release (b_2_) of diclofenac was related to the amphiphilic properties of polymer until 6 days without cytotoxic effect (b_3_,b_4_) [88] (copyright requested), * *p* < 0.05.

These technologies are particularly suitable to process natural polysaccharides (i.e., chitosan, alginates) because they do not negatively influence some relevant features—i.e., hydrophilicity, biocompatibility—crucial to control molecular release mechanisms. This is extremely important in the case of chemotherapeutic treatments where diffusion mechanisms driven by the matrix properties can finely address the drug (i.e., doxorubicin) into the target tissue, thus significantly reducing collaterals effects [132]. In this view, an interesting aspect concerns the opportunity to tune the release of active molecules via stimuli-responsive properties of the carrier (i.e., pH). Recently, it was demonstrated that core–shell microspheres of cellulose and chitosan fabricated via EFDs and layer-by-layer (LbL) techniques could change water absorption and swelling behaviour in response to environmental factors. In vitro tests performed in simulated gastric fluids (SGF) and simulated intestinal fluid (SIF) have highlighted an inhibition of the drug release in the presence of acidic conditions—typically present in the stomach—thus suggesting the carrier as a gastroprotective system for selective delivery of therapeutic agents along the intestine (Figure 7A) [86]. 

Other studies have recently proposed investigating amphiphilic materials to improve drug protection by modulating the interactions between hydrophilic and hydrophobic fragments. For instance, cellulose-grafted PCL nanoparticles composed of amphiphilic cellulose backbone and hydrophobic PCL grafts copolymer fabricated via electrospraying were investigated as a drug delivery system of anti-inflammatory drugs (i.e., diclofenac). It was demonstrated the interactions of diclofenac with cellulose fragments via H-bonding and polar ones with PCL hydrophobic chains concur to delay the diffusion of the molecules through the chain network, thus promoting a sustained release over 96 h (Figure 7B) [88]. 

At the early stages of tumour development, inflammatory processes are triggered, and influence on tumour development and progression associated with the chronic inflammation [134]. Plant-derived extracts are characterised by their anti-inflammatory effect; moreover, they have been used as a greener chemical approach for the biosynthesis of copper [135], silver [136], and gold [137] nanoparticles, which have been shown to mediate the ROS production, decrease the cytokines production, and prevent the inflammation via autophagia, respectively. 

### 5.2. Biomedical Applications: In Vitro 3D Models 

Biomaterial production is currently based on bio-fabrication methods that usually ‘build as the biology’—by an accurate assembly of living (i.e., cells) and not living (i.e., extracellular matter) products. In recent years, this conventional approach in the biomedical area rapidly matches the development of green technology firmly used to design bioinspired materials as alternative solutions to animal-based products used to fabricate bio-based food [13] and leather [14,71]. In addition, animal testing is widespread, especially in pharmaceutics research and development, and its increasing use shows a growing environmental impact. Introducing new technical practices based on preclinical in vitro models to minimise the use of in vivo studies contributes to a new idea of sustainability in this area [138].

In this view, novel bio-fabrication techniques for the fabrication of 3D bio-inspired platforms recently offered a great chance to more faithfully mimic physiological and biological characteristics of the in vivo environment to more accurately study the biological process of healthy and diseased tissues, thus predicting the effects of drug interaction in vivo [139,140].

**Figure 8:**
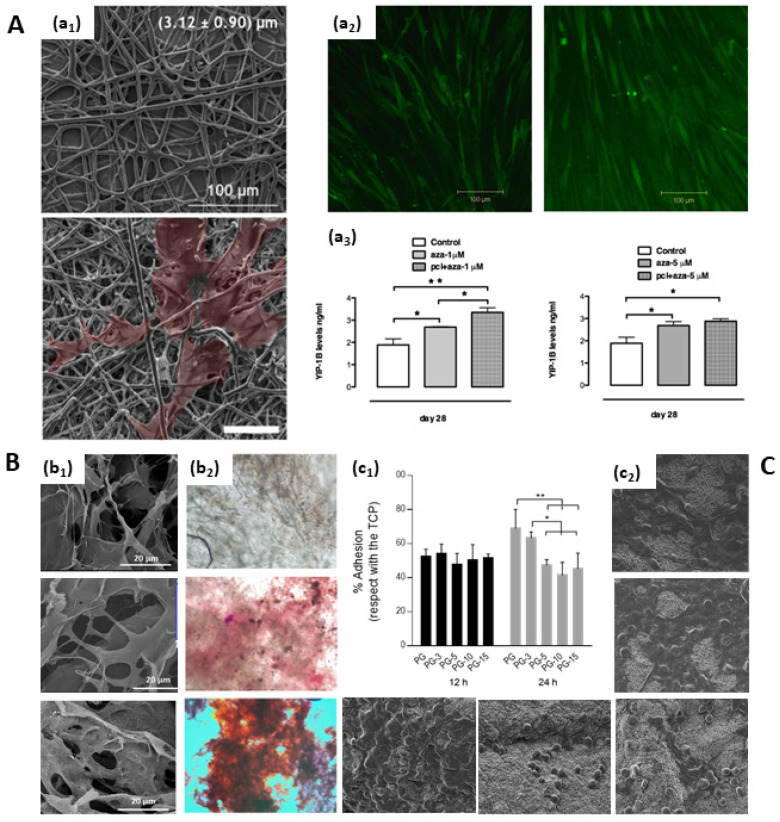
In vitro models for studying the effect of active molecules on cells. (**A**) 5-AZA treatment on FDA-approved electrospun scaffolds for skeletal muscle regeneration. (a_1_,a_2_) Cell morphology and (a_3_) quantitative effects on hMSC proliferation rate and differentiation [83] (copyright requested). (**B**) T-CNF-graft-SPH and T-CNF-graft-SPH/CaP: nanofibrils morphology (b_1_) with or (b_2_) without alizarin red staining to detect newly formed mineralised matrix by hMSCs [16] (Copyright requested); (**C**) Sulfonatocalixarene-loaded gelatin-based nanofibers: (c_1_) quantitative and (c_2_) qualitative studies on drug-mediated cell adhesion mechanisms [142]. (* *p* < 0.05, ** *p* < 0.01).

For instance, several studies have been focused on the design of micro- or nanofibers to be used as in vitro cell culture platforms to study the effect of released active molecules on cell behaviour. For instance, FDA-approved biodegradable nanofibers have been used as a preclinical model to evaluate the effect of active molecules, such as 5-azacytidine (5-AZA), that promote the upregulation of muscle genes [83]. In particular, nanofibers have been widely used to mimic the cellular niche. Moreover, results have also shown that electrospun fibres could mediate the activity of 5-AZA on hMSCs (Figure 8A). In this context, using materials from renewable sources such as cellulose and its derivates certainly concurs with developing substrates that follow the main principles of sustainability, eco-efficiency, and green chemistry. In this regard, 2,2,6,6-tetramethylpiperidine-1-oxyl (TEMPO)-mediated oxidation was optimised to produce TEMPO oxidised cellulose nanofibers (T-CNF), grafted with soy protein (SPH) (Figure 8B) [141]. It was demonstrated that green proteins such as soy could support biocompatibility, promoting efficient bio-mineralisation in the presence of simulated body fluids. Hence, it can work as an excellent 3D model to investigate the cell mechanism in mineralised matrix deposition during bone regeneration. Alternatively, innovative approaches to integrating supramolecular structures into fibrous matrices have been explored to fabricate innovative platforms for sustainable treatments. For instance, calixarenes loaded in gelatin-based nanofibers can be successfully used to neutralise the effects of high concentrations of iodine that usually produce critical ecological and health effects. Recent studies on in vitro response allowed us to explore the effect of calixerene on the adhesion of hMSC (Figure 8C) [142], remarking the potential use of drug-loaded nanofibers and as an innovative in vitro model to investigate the cell response under pharmacological treatments. 

Additionally, using protein-based nanofibers is raising wide interest in different areas as a function of the protein source—particularly mammalian gelatin for pharma and agrifood applications [143,144]. Gelatin-based nanofibers also provide biochemical signals required to trigger cell adhesion due to the presence of binding motifs as Arg–Gly–Asp (RGD) to form integrins natively present in the tissues [117,144]. The dissolution of gelatin in non-toxic solvents (i.e., acetic acid) facilitates the preservation of these chemical functionalities during the fibres’ processing, making the fabrication of biocompatible fibres for in vitro applications [145]. In this context, the use of other proteins from waste is growing rapidly to design in vitro models, including wool waste from the textile industry, or zein, a by-product of the corn wet-milling [146,147].

Recent studies have shown that wool keratin can improve cell interaction similarly to gelatin [126], while zein lacks bioactivity due to its hydrophilicity, which is preferable in pharmaceuticals [146]. 

In recent decades, cancer research has evolved from 2D cell cultures to animal models and, more recently, to 3D in vitro cancer models to study the mechanisms of cancer in a more controllable and sustainable manner avoiding animal models. The most used 3D in vitro models for cancer research are spheroids, fibre scaffolds, and bioreactors [147,148]. To replicate the 3D tumour microenvironment, two methods were used, the fabrication of hydrogel microspheres and cell-dense spheroids that were bio-assembled using an automated bio-fabrication process with high reproducibility to study tumourogenesis and drug delivery [149]. To study cancer biology and drug screening application in vitro, a facile freeze-drying process fabricated a 3D macroporous prepared by bacterial cellulose [150]. At the same time, water-soluble polymers, PVA, and gum arabic (GA) were used to fabricate electrospun fibres to predict the therapeutic success of GA-gold nanoparticles (GA-AuNPs) delivered to metastatic melanoma cells as a tool for precision medicine [151]. 

### 5.3. Nutraceutical Applications

In addition to pharmaceutical applications, another wide and well-explored application field of active agents is a nutraceutical. A definition of nutraceutical is based on the definition of Stephent. DeFelice is “any substance that is a food or part of a food and provides medical or health benefits, including the prevention and treatment of disease” [152].

Medicinal—i.e., used as a source of drugs—and aromatic—i.e., used for aroma and flavour—plant derivatives with Generally Recognized As Safe (GRAS) status are used in many areas in the food industry because they have many properties ranging from extending the shelf life of foods, up to add antioxidant or antimicrobial activity [153,154]. 

These active agents derived from plants can be used as spices, herbal tea, food supplements, and additives. The best-known aromatic plants originate from temperate and warm countries such as those in the Mediterranean, and tropical ones contain bioactive compounds with different compositions and activities. Examples of Mediterranean include oregano, rosemary, sage, anise, basil, among others, which have been used as extracts and essential oils because they contain many biologically active compounds, mainly polyphenolics [153]. 

Another plentiful source of active ingredients is microalgae, rapidly growing unicellular microorganisms in freshwater or saline water environments. They are rich in high-value nutrients and essential elements, including carbohydrates, amino acids, polyunsaturated fatty acids, vitamins, and natural pigments. Moreover, they accumulate essential elements such as potassium, zinc, iodine, selenium, iron, manganese, copper, phosphorus, sodium, nitrogen, magnesium, cobalt, molybdenum, sulphur, and calcium [155].

As already reported, a very rich source of active ingredients are honeybee colonies. In fact, all the substances obtained from them contain vitamins (A, C, and E), proteins (peptides and all essential amino acids), polysaccharides (cellulose, callose, glucan, lignin, and sporopollenin), lipids, minerals (i.e., Ca, Cu, Fe, K, Mg, and Na) that are very useful for nutraceuticals. Mixing these active ingredients may help to achieve food products with high nutritional quality for healthier diets and with potential medical activity (anti-inflammatory, anti-cancer, antioxidant, etc.) [156].

However, it is not easy to include active ingredients from medicinal and aromatic plants in food products. Food substances are a complex system of interconnected different microenvironments, and plant extracts often have low water solubility and high volatility. Moreover, they are easily affected by external factors such as light, oxidation, and heating, causing their rapid deterioration. Therefore, encapsulating these substances in vectors (i.e., micelles, particles, hydrogels) is necessary to preserve their activity and improve their effect [155].

To realize vectors for nutraceuticals encapsulation and delivery, it is possible to use proteins, polysaccharides, or lipids because they are abundant, sustainable, and non-toxic. Multiple edible delivery systems have been developed for nutraceutical applications, including particles, emulsions, films, and hydrogels, to improve the solubility of active ingredients, avoid interactions with the food matrix before consumption, and control their release [157].

For example, edible nano-emulsions loaded with coenzyme Q10 were prepared by microfluidics using a mixture of milk proteins, lecithin, and octenyl succinic anhydride-modified starch as surfactants. The behaviour of a high-protein beverage containing the loaded nano-emulsions was evaluated during in vivo gastrointestinal digestion. The reported results demonstrated the enhanced bioavailability and stability of Q10 coenzyme, notwithstanding the selected surfactants [158].

It is possible to use different technologies to realize a delivery system starting from an emulsion. It is reported that the preparation of curcumin-loaded particles starts from an oil/water emulsion of whey protein using spray drying (SD) and electrospraying (ES). Regarding morphological difference between the samples, ES permits spherical and nano-sized particles, whereas particles obtained by SD are larger, and surface depression and agglomeration are noticeable. The encapsulation efficiency of curcumin and dissolution behaviour is also different; it is higher for ES particles due to the morphology and process parameters [159].

Oil-in-water emulsion gels can be useful for encapsulating hydrophobic and hydrophilic active components by starting from food-grade ingredients—proteins and polysaccharides—using a relatively simple manufacturing process. For example, the advantages and disadvantages of the internal and external gelation mechanisms for creating bulk emulsion gels loaded with lycopene have been investigated. The microstructure, texture, water behaviour, and in vitro digestion of lycopene were analysed for both systems. The results showed that the internal gelation led to better results concerning the external one [160].

Complex systems have been studied for the encapsulation and delivery of active ingredients for the dual purposes of enhancing nutrient delivery and preserving perishable foods. First, lipid-based nanoparticles loaded with resveratrol were realized by sonication process using a mixture of soy phosphatidylcholine (PC), (+)-α-tocopherol acetate (αTA), (±)-α-tocopherol nicotinate (αTN), and a non-ionic surfactant (Tween 80). Then, nanoparticles were dispersed into low-molecular-weight chitosan. Ultimately, freestanding films were fabricated by solution casting on the strawberry surface. The composite films revealed excellent antimicrobial activity, reduced dehydration and texture deterioration of fruit, and increased the absorption of resveratrol by oral administration [161].

## 6. Conclusions and Future Trends 

The majority of manufacturing technologies for drug delivery systems currently involve the use of synthetic materials, with “biologically safe” responses but without a direct biological activity, which drastically limits success in the long term. Furthermore, drug delivery systems have been more recently fabricated by using naturally derived polymers to form bioactive or biomimetic carriers that can interact with the biological microenvironment, thus influencing cell activities during the pharmaceutical treatment, with potential benefits/limitations related to long-term side effects. 

In this context, the rapid evolution of traditional concepts of materials processing, mainly ascribable to the recent discoveries in green chemistry, is leading to the formulation of novel materials for drug delivery by sustainable routes with low cost, high availability, limited toxicity, and low environmental impact generated by the accumulation of industrial by-products. In this view, optimising green nanotechnologies able to size materials at the nanoscale currently represents the most relevant challenge to designing innovative drug delivery systems with distinctive and improved functionalities. The fabrication of nanoparticles combining small sizes and extended surface-area-to-volume ratio is relevant to customise fundamental parameters to control drug administration mechanisms, including molecular solubility, matrix chemical/physical and mechanical stability, and peculiar functionalities (i.e., electrical, optical, magnetic).

This opens up a new scenario for the fabrication of smart devices in different application areas of medicine/biomedicine (i.e., in vivo imaging, bio-sensing, molecular targeting, gene delivery, cancer therapy, and artificial implants) with significant benefits for industry, environment, and society, via the implementation of new simple approaches that are ecologically and economically more sustainable, towards people living healthier lives in a cleaner world.

## Figures and Tables

**Table 1:** Classification of sustainable polymers.

Sustainable Polymers			
Natural Sources		Synthetic Sources	
*Animals*	*Plants*	*Biomasses*	*Bio-Monomers*
Gelatin	Starch	PLA [43]	PBS [44]
Albumin	Cellulose	PHB [45]	Polyamides [46]
Collagen	Hemicellulose	PHBV [47]	PE [48]
Hyaluronic acid	Lignin		PET [49]
Keratine	Alginate		Epoxy resins [50]
Silk fibroin	Zein		Elastomers [51]
Chitosan			

## Data Availability

Not applicable.
